# Lighting the wick in the candle of learning: generating a prediction stimulates curiosity

**DOI:** 10.1038/s41539-019-0056-y

**Published:** 2019-10-21

**Authors:** Garvin Brod, Jasmin Breitwieser

**Affiliations:** 1 DIPF | Leibniz Institute for Research and Information in Education, Frankfurt am Main, Germany; 2 Department of Psychology, Goethe University Frankfurt, Frankfurt am Main, Germany

**Keywords:** Human behaviour, Human behaviour, Education

## Abstract

Curiosity stimulates learning. We tested whether curiosity itself can be stimulated—not by extrinsic rewards but by an intrinsic desire to know whether a prediction holds true. Participants performed a numerical-facts learning task in which they had to generate either a prediction or an example before rating their curiosity and seeing the correct answer. More facts received high-curiosity ratings in the prediction condition, which indicates that generating predictions stimulated curiosity. In turn, high curiosity, compared with low curiosity, was associated with better memory for the correct answer. Concurrent pupillary data revealed that higher curiosity was associated with larger pupil dilation during anticipation of the correct answer. Pupil dilation was further enhanced when participants generated a prediction rather than an example, both during anticipation of the correct answer and in response to seeing it. These results suggest that generating a prediction stimulates curiosity by increasing the relevance of the knowledge gap.

## Introduction

“Curiosity is the wick in the candle of learning.” This well-known quote by William Arthur Ward^1^ deftly depicts curiosity as a driving force for learning, akin to a wick that determines how and for how long a candle burns. Curiosity, also called epistemic curiosity in the context of knowledge acquisition, can be broadly defined as the desire to know or learn something in the absence of extrinsic rewards (for more in-depth discussions about defining curiosity, see refs. ^2–4^). Although psychologists have been interested in the psychological and biological mechanisms that give rise to curiosity for a long time^5^ (for an overview, see ref. ^6^), only fairly recently has it been demonstrated that greater curiosity is indeed associated with better learning.

A number of studies have shown that facts about which participants have indicated they have high curiosity are better remembered than facts about which participants have indicated they have low curiosity.^7–11^ Studies using pupillometry and functional magnetic resonance imaging (fMRI) to investigate this association have suggested neural mechanisms by which curiosity promotes learning. Kang et al.^11^ demonstrated that curiosity is accompanied by a dilation of the pupil, a marker for the release of the neurotransmitter noradrenaline in the brainstem’s locus coeruleus.^12^ The release of noradrenaline leads to an upregulation of the sensitivity of cortical processing units, thus facilitating learning.^13^ In addition, fMRI studies indicate that activation of brain regions involved in successful memory encoding and reward anticipation is enhanced when curiosity is high.^7,11,14–16^ In summary, research suggests that curiosity enhances learning by upregulating neural processing, particularly in brain regions involved in successful memory encoding.

Can curiosity be stimulated? In early work on this topic, Berlyne^5^ speculated that when a learner is asked a question that he or she cannot answer, this should result in curiosity and, hence, arousal. When the correct answer is revealed, the resulting reduction of arousal should lead to better learning. He suggested that new information should therefore be presented to learners in the form of questions for which the learners have to try to predict the answers. These notions are consistent with the prominent information-gap proposal,^6^ according to which learners become curious when their attention becomes focused on a gap in their knowledge. Loewenstein^6^ also argued that guessing with feedback stimulates curiosity because it increases the salience of the knowledge gap. In line with these ideas, a number of studies have demonstrated that guessing with feedback promotes learning.^17–20^ These studies did not assess curiosity, however, so it is unclear whether the observed benefit arose because of enhanced curiosity or even whether curiosity was enhanced at all.

Predicting an answer before being told the correct one could benefit learning for reasons other than enhanced curiosity (for a detailed discussion, see ref. ^21^). First, to generate a prediction, learners have to retrieve prior knowledge and connect it to the new information being learned, which fosters meaningful learning.^22^ Second, when learners generate a prediction, they are first presented with a problem. Information presented as a problem is more likely to be used later on for solving similar problems than is information presented in the form of simple facts.^23^ Third, the pupillary surprise reaction to expectancy-violating information is enhanced when learners make a prediction beforehand, which also boosts their memory.^21^ In conclusion, initial evidence suggests that generating predictions is an effective instructional method that promotes learning. It is unclear, however, whether generating predictions derives part of its effectiveness from stimulating curiosity.

In the current study, we tested whether generating a prediction before seeing the correct answer to a question stimulates curiosity for numerical trivia facts. The prediction condition was compared with a closely matched control condition in which participants had to generate a relevant example before seeing the answer. Generating examples is also an effective-learning strategy that entails retrieving prior knowledge; in addition, it fosters deep encoding of new information.^24^ We hypothesized that more facts would receive high-curiosity ratings in the prediction condition than in the control condition, and that higher curiosity would be associated with better memory for the facts. We further tested whether the pupil dilation response was a sensitive marker of the buildup and relief of curiosity and whether it was amplified in the prediction condition.

## Results

### Behavioral data

**Fig. 1:**
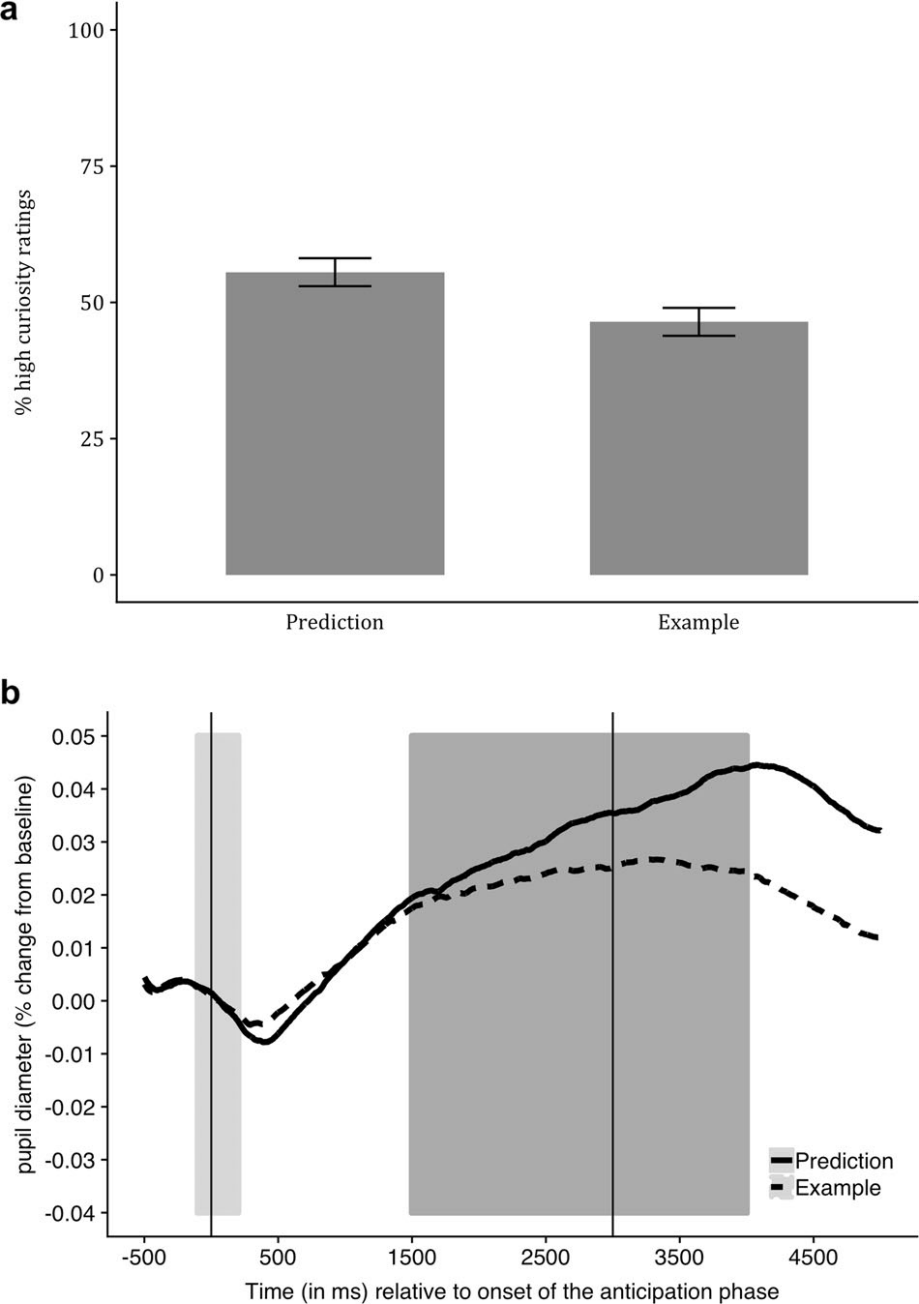
Generating a prediction enhances curiosity. **a** More facts received high-curiosity ratings in the prediction condition than in the example condition. Error bars represent within-subject standard error. **b** The average pupillary response during the anticipation phase was greater in the prediction condition than in the example condition. The average percentage change in pupil diameter relative to the baseline (light gray area) was calculated for the time interval from 1.5 to 4 s after the onset of the anticipation phase (dark gray area)

As we hypothesized, more facts received high-curiosity ratings in the prediction condition (*M* = 55.55, *SD* = 14.36) than in the example condition (*M* = 46.43 and *SD* = 15.96), *t*(28) = 1.78, *p* = 0.043, and *d* = 0.330 (Fig. 1a). Similarly, absolute curiosity ratings were significantly higher in the prediction condition (5.57 ± 1.53) than in the example condition (5.13 ± 1.31), *t*(28) = 2.21, *p* = 0.018, and *d* = 0.411. Also as we expected, high-curiosity facts (*M* = 61.95 and *SD* = 17.63) were better remembered than low-curiosity facts (*M* = 58.21, *SD* = 16.64), *t*(28) = 1.76, *p* = 0.044, and *d* = 0.327. It took participants longer to generate a prediction (6.61 ± 2.21 s) than to generate an example (5.51 ± 1.49 s), *t*(28) = 3.63, and *p* = 0.001. Memory performance did not differ significantly between the two conditions, however (prediction: *M* = 60.42 and *SD* = 15.81; example: *M* = 59.52, *SD* = 16.78), *t*(28) = 0.41, *p* = 0.682, and *d* = 0.077. We also explored whether the effect of curiosity on memory performance was qualified by an interaction between curiosity and condition. A within-subjects ANOVA revealed no interaction, *F*(1, 28) = 0.70, *p* = 0.411, and *η*_p_ = 0.024.

Table 1 shows the results for the questionnaire data. In short, participants’ ratings were in accordance with their performance in that they thought they had been more curious in the prediction condition than in the example condition, *t*(28) = –3.62 and *p* = 0.001, but they were undecided about in which condition they had learned more, *t*(28) = –1.32 and *p* = 0.199. In addition, participants reported that they had more often connected the facts with prior knowledge in the example condition, *t*(28) = 3.08 and *p* = 0.005, but that they thought more often about their predictions than about their examples during the memory test, *t*(28) = 2.48 and *p* = 0.019.

**Table 1:** Results for the questionnaire Items

	Mean	Median	*SD*
In which condition were you more curious?^a^	2.41	2	1.62
In which condition did you learn more?^a^	3.14	3	1.48
Prediction condition			
Did you connect the facts to your prior knowledge during learning?^b^	3.69	4	0.89
Did you compare the correct value with your predictions?^b^	4.38	5	0.86
Did you think of your predictions during the memory test?^b^	4.14	4	0.64
Example condition			
Did you connect the facts to your prior knowledge during learning?^b^	4.14	4	0.69
Did you connect the results with your examples?^b^	3.24	3	0.99
Did you think of your examples during the memory test?^b^	3.52	4	1.38

^a^Responses to these questions were on a scale from 1, *clearly prediction*, to 6, *clearly example*

^b^Responses to these questions were on a scale from 1, *never*, to 5, *always*

### Pupillary data

**Fig. 2:**
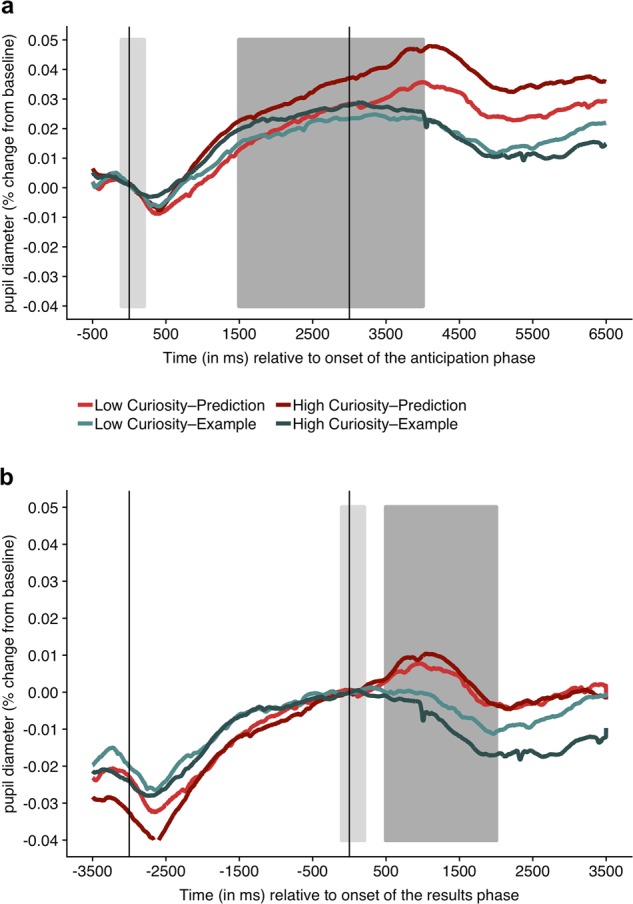
Pupil dilations as a measure of curiosity and surprise. **a** During anticipation of the answer, high-curiosity facts induced greater pupil dilation than low-curiosity facts. **b** In response to seeing the answer, pupil dilation was greater in the prediction condition than in the example condition, indicating that generating a prediction promotes surprise, but there was no effect of curiosity. The two graphs depict the pupillary time series during the same time window but differ in the baseline windows (beige areas) used to calculate the percentage change in pupil diameter. The baseline was placed around the onset of the anticipation phase for the time series in **a** and around the onset of the results phase for the time series in **b**, to ensure that the effects of curiosity in the results phase were not confounded by the preceding pupil dilation during the anticipation phase. Average percentage change of pupil diameter was calculated separately for the two analysis windows (gray areas)

We first examined the pupillary response during the anticipation phase, before participants saw the correct answer, which is when curiosity should have been maximal. As the pupillary time series in Fig. 1b shows, the average pupillary response during the anticipation phase was greater in the prediction condition than in the example condition, *t*(28) = 2.24, *p* = 0.016, and *d* = 0.417. We also tested whether the pupillary response during the anticipation phase was modulated by curiosity (Fig. 2a) and found that high-curiosity facts induced greater pupil dilation than low-curiosity facts in the prediction condition, *t*(28) = 1.93, *p* = 0.032, and *d* = 0.357. This effect was marginally significant in the example condition as well, *t*(28) = 1.66, *p* = 0.054, and *d* = 0.308.

In addition to performing these preregistered analyses, we explored the pupillary response to seeing the correct answer as a proxy for surprise (see ref. ^21^) and whether it is modulated by curiosity (Fig. 2b). A within-subjects ANOVA revealed a main effect of condition, *F*(1, 28) = 24.18, *p* < 0.001, and *η*_p_ = 0.463; pupil dilation was greater in the prediction condition than in the example condition, indicating that generating a prediction promotes surprise. There was no main effect of curiosity, *F*(1, 28) = 0.34, *p* = 0.567, and *η*_p_ = 0.012. However, this null effect was qualified by a significant Curiosity × Condition interaction, *F*(1, 28) = 4.24, *p* = 0.049, and *η*_p_ = 0.132. Follow-up post-hoc tests were nonsignificant, however, rendering the interpretation of the interaction inconclusive.

## Discussion

This study revealed that asking learners to generate a prediction about a numerical fact stimulates their curiosity about the true number. More facts received high-curiosity ratings when learners predicted the correct answer than when they had to generate an example. Greater self-rated curiosity was, in turn, associated with better memory for the true number. Overall memory performance did not differ between conditions, however. Concurrent pupillary data revealed that high curiosity, compared with low curiosity, was associated with larger pupil dilation during anticipation of the correct answer. Pupil dilation was further enhanced when participants generated a prediction, both during anticipation of the correct answer and in response to seeing it.

How could generating a prediction stimulate curiosity? Berlyne^5^ hypothesized that generating a prediction leads to increased arousal, which is relieved when learners see the correct answer. Loewenstein^6^ argued that prediction plus feedback stimulates curiosity because it increases the learner’s attention to his or her knowledge gaps. These conjectures are not mutually exclusive; indeed, our pupillary data could be interpreted as providing support for both by showing that generating a prediction leads to a larger pupil dilation response during anticipation of the correct answer as well as in response to seeing it. Based on these theories, we interpret our findings to suggest that generating a prediction increases the relevance of the knowledge gap, i.e., the subjective value of the soon-to-be-learned information. The increased relevance then plays out in enhanced arousal in anticipation of the solution as well as in enhanced attention to the knowledge gap. As outlined in the next paragraph, we speculate that a key driver of the increased curiosity is that participants committed to an outcome before seeing the correct one, which is inherent in generating a prediction.

Potts et al.^25^ (Experiments 3 and 4) recently demonstrated that guessing the translation of a foreign language word before seeing the correct translation leads to greater self-rated curiosity than when no guess was required or when guessing was done after the curiosity rating. These findings indicate that the act of generating a response can lead to increased curiosity. In the current study, we compared two conditions in which participants had to generate a response in each before rating their curiosity. Our findings, thus, suggest that there is more to the curiosity-enhancing effect of generating a prediction than just generating a response. We speculate that it is the act of a priori committing to a specific outcome, which is inherent in generating a prediction, that leads to the observed boost in curiosity that goes beyond the benefit of generating a response. Committing to a specific outcome might increase the relevance of the knowledge gap by raising awareness of the gap (i.e., knowing that you do not know the correct number) and by increasing anticipation of the feedback, which is in line with the observed increased pupil dilation during anticipation of the correct answer in the prediction condition.

The “increased relevance” interpretation is also in line with the observed increased pupil dilation in response to seeing the correct answer in the prediction condition, which suggests that generating a prediction promotes surprise (see ref. ^21^). Again, the current study, which compared two generative-learning conditions, suggests that the act of generating is not sufficient to elicit surprise. Rather, a priori committing to a specific outcome seems necessary for surprise to occur.

Our results further indicate that curiosity is closely linked to pupil dilation and, thereby, to noradrenergic activity. During anticipation of the correct answer, pupil dilation was greatest when participants had both reported high curiosity about a fact and generated a prediction regarding the correct answer, and pupil dilation was smallest when participants had reported low curiosity about a fact and had not generated a prediction. This pattern suggests that the amount of pupil dilation is a sensitive measure of the strength of curiosity. Pupil dilation is preceded by the release of noradrenaline,^26^ and noradrenergic activity modulates attentional focus and boosts processing of task-relevant information.^12,13^ Together, these observations suggest that curiosity promotes learning by upregulating neural processing, thus preparing the brain for subsequent learning.

Choosing the right control condition is the linchpin of experimental research. We evaluated the effects of generating a prediction relative to a control condition in which participants generated an example. We chose generating examples because this activity could be closely matched to generating predictions; it was performed prior to the curiosity rating and also entailed activation of prior knowledge. Such activation was important so that we could exclude alternative explanations for the curiosity boost (e.g., the possibility that any generative learning activity could stimulate curiosity). However, this design choice might also have worked against us. According to the knowledge-gap proposal,^6^ activating prior knowledge could suffice to make knowledge gaps apparent, which would result in enhanced curiosity not only in the prediction condition but also in the example condition. An additional factor that likely worked against observing a difference between the prediction and examples conditions was that, given our interest in pupil dilation, we used an identical 3-s anticipation phase prior to the presentation of the correct answer in the two conditions. This means that participants had some time to implicitly generate a hypothesis in the example condition as well. Thus, our experiment was a conservative test of the effects of generating predictions on curiosity.

As in previous research, curiosity was positively related to later memory for the facts presented. However, although generating predictions rather than examples led to higher curiosity, generating predictions was not per se more effective in promoting learning. Responses to the questionnaire suggest some hypotheses about this apparent contradiction. Participants reported that they had connected the facts with prior knowledge better in the example condition than in the prediction condition, which likely compensated for the benefit afforded by curiosity in the prediction condition. On the whole, our findings suggest that generating predictions and generating examples derive their effectiveness from common as well as unique cognitive mechanisms. Although our study indicates that stimulating curiosity is worthwhile because it promotes learning, it does not suggest that curiosity is the only factor that determines learning performance.

We see several limitations to our study. The first is the short time span between the study and test phases. This resulted from our primary focus on how generating predictions could stimulate the buildup of curiosity during learning. However, this aspect of our procedure precludes drawing conclusions about whether the beneficial effects of curiosity on memory increase, decrease, or even vanish in the longer term. This is an as-yet unanswered question that deserves attention in future research. A second limitation is the sample size, which was too small for individual differences analyses. Sample size was determined a priori with a view to testing within-subjects condition differences, not between-subjects correlations. It would be fruitful to investigate individual differences in curiosity and how they relate to learning in future studies. A third limitation is the study material: simple, isolated facts. Although such stimuli are commonly used in research on curiosity and learning, they are not typical of the content taught in schools and universities. There is, thus, a need to replicate our findings using more complex stimuli, such as scientific concepts. A final limitation is that our study cannot provide causal evidence for the effects of feedback on curiosity (or for the combination of prediction and feedback as argued by Loewenstein^6^) because our curiosity rating always took place before the feedback was presented. However, participants anticipated feedback while making the curiosity rating because they were always given one afterwards. It is an open question whether guessing without anticipated feedback would boost curiosity as well.

If curiosity is indeed the wick in the candle of learning, how can it be lit? Finding instructional strategies that reliably stimulate curiosity in learners could be a major contribution of the learning sciences to educational practice. The results of this study indicate that a good strategy is to ask learners to generate a prediction before telling them the correct answer to a question. In line with the definition of curiosity, this boost is not achieved through extrinsic rewards but through an intrinsic desire to know whether a prediction holds true or not. Generating a prediction is also an efficient instructional strategy^21^; it can be used quickly, repeatedly, and simultaneously within a large group of students, and with the help of ubiquitous mobile devices such as smartphones or tablets. Generating predictions is already a part of successful instructional curricula, in particular in the sciences.^27,28^ Our study suggests that these curricula derive part of their success from stimulating curiosity.

## Methods

This study (including the main hypotheses, sampling plan, design, and analysis plan) was preregistered on the Open Science Framework (https://osf.io/hjr5k).

### Participants

The participants were 33 university students (mean age = 22.97; 22 female) who were native speakers of German. The data of four participants were discarded, in two cases because of technical problems, in one case because the participant’s age fell far outside the age range in the preregistered plan, and in one case because of a lack of variance in the curiosity ratings, i.e., rating of 1 on a scale from 1 to 10 on 79% of trials. We had not anticipated the latter situation in our preregistered plan and noticed the inadequate use of the curiosity scale by this participant only during analyses. We confirmed that including this participant would not have altered our main results. All reported analyses were conducted with the remaining data set from 29 participants (mean age = 22.28, range = 19–31; 19 female). Participants were recruited through bulletins within the university community and gave written informed consent prior to testing. The sample size was determined a priori using G*Power 3.1^29^ with the following settings: paired *t* test (one-tailed), *d*_z_ = 0.55, *α* = 0.05, *β* = 0.90. Participants were paid €10 or received course credit for their participation. Ethics approval was obtained from the ethics committee of the DIPF | Leibniz Institute for Research and Information in Education.

### Design

We used a within-subjects design with one independent variable (condition) with two levels (prediction and example). The dependent variables for the behavioral analyses were participants’ curiosity ratings and memory performance in the test phase.

### Stimuli

Ninety numerical facts in the form of “percentage of” were drawn from sources such as the Federal Statistical Office. The facts were worded to fit the format “X out of 10” (the values of X were rounded as needed); none of the correct answers were “0” or “10.” The set of 90 facts was divided into two subsets of 45 facts each. The assignment of the two subsets to the two conditions was counterbalanced across participants, whereas the order of items remained the same within each subset list.

### Procedure

We administered the numerical-facts learning task as a fully-computerized experimental task. Each condition (prediction and example) consisted of a study phase followed by a test phase. The order of conditions was counterbalanced across participants. Testing for order effects by including order of conditions as an additional factor did not yield any significant interactions. Each study phase started with two practice trials, followed by 45 facts. Participants were instructed to remember the facts for the subsequent memory test.

**Fig. 3:**
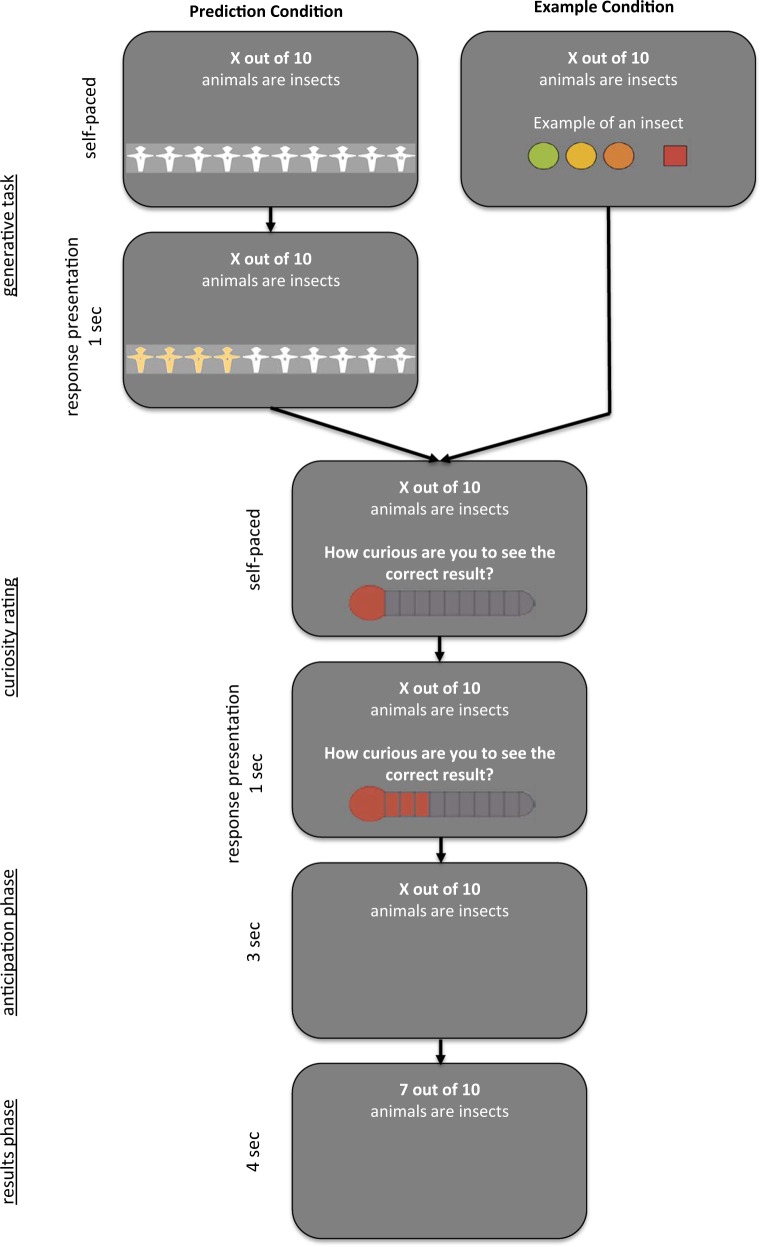
Schematic overview of the study phase. The two experimental conditions differed only in the generative task at the beginning of each trial: participants had to generate either a prediction for the correct value of X (prediction condition) or an example relevant to the fact (example condition). After this task, they rated their curiosity about the answer on a 10-point visual analog scale (portrayed as a stylized thermometer). Next, they saw the initial question again during a brief delay and finally were shown the correct number

In the study phases of both conditions (Fig. 3), participants were presented with a series of incomplete facts in “X out of 10” format; (the X was a placeholder for a missing value). Following the presentation of each fact, they rated their curiosity about the missing value on a 10-point visual analog scale (1 = lowest curiosity, portrayed as a stylized mercury-filled thermometer). After a brief delay in which they saw the initial question again (the anticipation phase), they were shown the correct number. The two conditions differed in the generative activity that took place before the curiosity rating. That is, in the prediction condition, participants were asked to report their prediction for the correct number by clicking on a 10-point visual analog scale (portrayed as a series of ten manikins). The response was then highlighted for 1 s. In the example condition, instead of predicting the correct number, participants were asked to generate an instance relevant to the fact. They were instructed to click on one of three colored buttons as soon as they had thought of an example or to click on the red square if they could not think of one. Participants indicated the perceived difficulty of generating an example (low, medium, and high) through their choice of which colored button to click on. In our analyses, we explored whether this difficulty rating was associated with curiosity and memory performance. Participants did not state their examples aloud, but to ensure compliance with the task, we told them beforehand that they would be asked to provide their examples after completion of the task. To follow up on this announcement, we later asked them to provide the examples they generated for a sample of ten prespecified items.

The test phase was preceded by a filler task (~1 min), in which participants repeated sequences of numbers in reversed order (digit-span-backward task). This task was intended to ensure that the facts were cleared from participants’ short-term memory. In addition, participants filled out a brief questionnaire in which they indicated (on a scale from 1, *never*, to 6, *always*) how often they had linked the facts to their prior knowledge during the study phase and how often they had compared the correct values with their predictions (or linked them to their examples in the example condition). The test phases were identical for the two conditions. Participants saw each of the 45 facts again and had to indicate the correct value of X on the visual analog scale. Responses were highlighted for 1 s before the start of the next trial.

Upon completion of the numerical-facts learning task, participants filled out a brief questionnaire in which they were asked (a) which facts they had known prior to the experiment, (b) whether they had been more curious in the prediction condition or the example condition, (c) whether they thought they learned more in the prediction condition or the example condition, and (d) whether they had thought about their predictions and examples during the memory test.

### Stimulus presentation and eye tracking

Stimuli were presented using PsychoPy 1.8.^30^ Participants were seated about 68 cm from the computer screen in a dimly lit room. The eye-tracking apparatus (EyeLink 1000, SR Research, Osgoode, ON, Canada) was located below the screen and recorded data continuously throughout the study phases at a frequency of 500 Hz. The purpose of the eye tracking was to record changes in participants’ pupil size during the anticipation phase, before they were shown the correct answer (Fig. 3).

### Data analyses

Data were analyzed using R.^31^ We used an *α* level of 0.05 in all the analyses. Across the prediction and example conditions, we discarded a trial if the participant had known the fact in question prior to the experiment. This was rarely the case, however (*M* = 0.38% of trials, range = 0–3.33%). We also discarded trials in the example condition if the participant did not think of an example (*M* = 3.75% of trials, range = 0–20%). Including these trials did not alter the reported condition effects, however.

The performance data were analyzed as specified in our preregistered analysis plan (https://osf.io/hjr5k). We specified only a rough analysis plan for the pupillary data, detailing the contrasts and statistical tests to be performed but not the time window. We did this because the exact time course of the pupillary response during the anticipation of the correct answer (i.e., when curiosity should have been maximal) could have differed from what Kang et al.^11^ observed given that the length of the anticipation phase differed between their study and ours. As we describe in detail below, the time courses were in fact highly similar, and we defined windows based on their data. Nevertheless, these analyses have to be considered exploratory.

#### Analysis of the performance data

Using Kang et al.’s^11^ approach, we analyzed participants’ curiosity ratings by sorting trials into high- and low-curiosity categories according to a participant-specific criterion (i.e., correcting for a participant’s overall ratings of curiosity). Our analysis differed from that of Kang et al.^11^ however, in that we used a trichotomization instead of a median split. That is, we computed the 33.33% quantile and the 66.67% quantile of each participant’s curiosity ratings across conditions. The trichotomization was chosen instead of a median split because it provides more distinct categories of high and low curiosity. Trials with a curiosity rating less than or equal to the participant’s 33.33% quantile were categorized as “low,” and trials with a curiosity rating greater than or equal to the participant’s 66.67% quantile were categorized as “high.” We excluded trials that fell in both categories or in neither category. We then determined the percentage of facts categorized as “high curiosity” in each condition. We exploratively compared this preregistered categorization approach to using the raw (absolute) curiosity ratings instead, which yielded similar results (see “Results” section). Memory performance during the test phase was assessed as the percentage of items for which the correct number was recalled exactly.

We also explored the difficulty of generating examples with a view to testing whether difficulty was associated with curiosity and memory performance. Because the majority of examples were rated as low difficulty (*M* = 82.72%, range = 40.00–97.78%, and *SD* = 13.10%), we recoded perceived difficulty into two categories (low vs. medium/high). Low difficulty ratings were given faster (*M* = 4.71 s and SD = 1.17) than medium or high difficulty ratings (*M* = 10.51 s and SD = 5.09) (*t*(28) = 6.84 and *p* < 0.001). Neither curiosity, *t*(28) = 1.07, *p* = 0.295, and *d* = 0.198, nor memory performance, *t*(28) = –0.49, *p* = 0.629, and *d* = 0.091, differed between difficulty levels, so we dropped the difficulty data from further analyses.

Paired *t* tests were performed to test (a) whether more facts were categorized as “high curiosity” in the prediction condition than in the example condition (one-tailed test), (b) whether memory performance was better for facts categorized as “high curiosity” than for facts categorized as “low curiosity” (one-tailed test), and (c) whether memory performance differed between the prediction and the example conditions (two-tailed test). In accordance with the preregistration, we performed the latter test as a nondirectional test because both generating predictions and generating examples are effective learning strategies and curiosity is only one factor determining learning success, which left it an open question whether the two conditions should differ in overall memory accuracy. Finally, a repeated measures ANOVA was computed to see if the effect of curiosity on memory performance was qualified by an interaction between curiosity and condition; that is, we hypothesized that curiosity might have a stronger impact on memory in the prediction condition than in the example condition.

#### Analysis of the pupillary data

Pupillary data were analyzed using itrackR (https://github.com/jashubbard/itrackR), an R package for high-level analysis of eye-tracking data, along with self-developed analysis scripts. Pupillary and behavioral data were merged, and the pupil data were aligned relative to the onset of the anticipation phase. Blinks were removed and the missing values were interpolated using cubic spline interpolation. Then, the pupillary data were normalized by subtracting the diameter at each time point from the average diameter across the period from 100 ms before the onset of the anticipation phase until 200 ms after the onset. Finally, the normalized values were divided by that average value. The resulting percentage-change measure was unconfounded from any nonspecific effect (e.g., effects of arousal or fatigue) that lasted longer than an individual trial.

To establish a marker of curiosity in the pupillary data, we calculated the average percentage change in pupil diameter for each participant 1.5 through 4 s after the onset of the anticipation phase (i.e., from 1.5 s before the onset of the results phase until 1 s after the onset of the results phase). This time window was chosen on the basis of the time course observed by Kang et al.^11^ who noted a ramp-up in pupil diameter about 1.5 s before the answer was displayed, and a drop back to baseline at around 1.5 s after the onset of this display. This assumed pupillary trajectory is consistent with the general idea that the pupillary response is sluggish (~1-s delay) and curbed to a frequency range below 4 Hz.^32^ We calculated average percentage change separately for the high-curiosity and low-curiosity trials, and separately for the prediction and example conditions. As outlined in our preregistered plan, we performed dependent-samples *t* tests on the pupillary data to determine the statistical significance of the difference between high-curiosity and low-curiosity trials in each condition and to test for differences between the conditions.

Given a previous finding that seeing the correct answer after making a prediction induces a pupil dilation response,^21^ we also explored the pupillary response to seeing the correct answer and whether it was modulated by curiosity. We calculated the average percentage change from baseline in pupil diameter from 0.5 to 2 s after the onset of the results phase, separately for each condition for each participant. This time window was chosen to be relatively earlier and shorter than the one used for the curiosity analyses because we were interested in the initial reaction to seeing the correct answer, whereas curiosity should ramp-up slowly in anticipation of the answer. To ensure that pupil dilation upon seeing the correct result was not confounded with the preceding pupil dilation during the anticipation phase, we chose a baseline phase around the onset of the results phase (100 ms before to 200 ms after onset). Given the exploratory nature of these analyses, average percentage change in pupil dilation was subjected to a repeated measures ANOVA omnibus test with curiosity (high and low) and condition (prediction and example) as within-subjects factors.

### Reporting summary

Further information on research design is available in the Nature Research Reporting Summary linked to this article.

## Supplementary information

Reporting Summary

## Data Availability

All data and materials, along with the preregistration, have been made publicly available via the Open Science Framework and can be accessed at https://osf.io/vpes5/.

## Supplementary information

**Supplementary Information** accompanies the paper on the *npj Science of Learning* website (10.1038/s41539-019-0056-y).

## Code availability

All analysis scripts have been made publicly available via the Open Science Framework and can be accessed at https://osf.io/vpes5/.
