# Supplementary material for: Lighting the wick in the candle of learning: generating a prediction stimulates curiosity
Source: NPJ Sci Learn. 2019 Oct 21;4:17. doi: 10.1038/s41539-019-0056-y (PMC6803639; doi:10.1038/s41539-019-0056-y)
Supplement: Supplementary file 1 — Reporting Summary [file 41539_2019_56_MOESM1_ESM.pdf]

nature research

Corresponding author(s): Garvin Brod

Last updated by author(s): May 29, 2019

Reporting Summary

Nature Research wishes to improve the reproducibility of the work that we publish. This form provides structure for consistency and transparency in reporting. For further information on Nature Research policies, see Authors & Referees and the Editorial Policy Checklist .

Statistics

For all statistical analyses, confirm that the following items are present in the figure legend, table legend, main text, or Methods section.

n/a	Confirmed	
☐	☒	The exact sample size (n) for each experimental group/condition, given as a discrete number and unit of measurement
☐	☒	A statement on whether measurements were taken from distinct samples or whether the same sample was measured repeatedly
☐	☒	The statistical test(s) used AND whether they are one- or two-sided<br>*Only common tests should be described solely by name; describe more complex techniques in the Methods section.*
☐	☒	A description of all covariates tested
☐	☒	A description of any assumptions or corrections, such as tests of normality and adjustment for multiple comparisons
☐	☒	A full description of the statistical parameters including central tendency (e.g. means) or other basic estimates (e.g. regression coefficient) AND variation (e.g. standard deviation) or associated estimates of uncertainty (e.g. confidence intervals)
☐	☒	For null hypothesis testing, the test statistic (e.g. F, t, r) with confidence intervals, effect sizes, degrees of freedom and P value noted<br>*Give P values as exact values whenever suitable.*
☒	☐	For Bayesian analysis, information on the choice of priors and Markov chain Monte Carlo settings
☒	☐	For hierarchical and complex designs, identification of the appropriate level for tests and full reporting of outcomes
☐	☒	Estimates of effect sizes (e.g. Cohen's d, Pearson's r), indicating how they were calculated

Our web collection on statistics for biologists contains articles on many of the points above.

Software and code

Policy information about availability of computer code

Data collection	PsychoPy2, EyeLink 1000 Eyetracker
Data analysis	Custom R code that is publicly available here: https://osf.io/vpes5/

For manuscripts utilizing custom algorithms or software that are central to the research but not yet described in published literature, software must be made available to editors/reviewers. We strongly encourage code deposition in a community repository (e.g. GitHub). See the Nature Research guidelines for submitting code & software for further information.

Data

Policy information about availability of data

All manuscripts must include a data availability statement. This statement should provide the following information, where applicable:

- Accession codes, unique identifiers, or web links for publicly available datasets
- A list of figures that have associated raw data
- A description of any restrictions on data availability

All data and materials, along with analysis scripts, have been made publicly available via the Open Science Framework and can be accessed at https://osf.io/vpes5/.

Field-specific reporting

Please select the one below that is the best fit for your research. If you are not sure, read the appropriate sections before making your selection.

☐ Life sciences ☒ Behavioural & social sciences ☐ Ecological, evolutionary & environmental sciences

For a reference copy of the document with all sections, see nature.com/documents/nr-reporting-summary-flat.pdf

Behavioural & social sciences study design

All studies must disclose on these points even when the disclosure is negative.

Study description	Quantitative experimental study
Research sample	The participants were 33 university students (mean age = 22.97; 22 female) who were native speakers of German.
Sampling strategy	The sample size was determined a priori using G*Power 3.1 24 with the following settings: paired t test (one-tailed), dz = 0.55, = .05, = .90. Participants were paid €10 or received course credit for their participation (convenience sample).
Data collection	The experiment was fully computerized (stimuli were presented using PsychoPy2). Eye-tracking was performed using an EyeLink 1000. The eye-tracking camera was positioned below the screen.
Timing	Data collection was performed between February 26 and June 7 2018.
Data exclusions	The data of four participants were discarded, in two cases because of technical problems, in one case because the participant's age fell far outside the age range in the preregistered plan, and in one case because of a lack of variance in the curiosity ratings (i.e., rating of 1 on a scale from 1 to 10 on 79% of trials . We had not anticipated the latter situation in our preregistered plan and noticed the inadequate use of the curiosity scale by this participant only during analyses. We confirmed that including this participant would not have altered our main results.
Non-participation	No participants declined participation or dropped out during the study.
Randomization	There is only one group because the experiment was performed fully within-subjects (order of conditions was counterbalanced pseudorandomly across participants).

Reporting for specific materials, systems and methods

We require information from authors about some types of materials, experimental systems and methods used in many studies. Here, indicate whether each material, system or method listed is relevant to your study. If you are not sure if a list item applies to your research, read the appropriate section before selecting a response.

Materials & experimental systems

n/a	Involved in the study	
☒	☐	Antibodies
☒	☐	Eukaryotic cell lines
☒	☐	Palaeontology
☒	☐	Animals and other organisms
☐	☒	Human research participants
☒	☐	Clinical data

Methods

n/a	Involved in the study	
☒	☐	ChIP-seq
☒	☐	Flow cytometry
☒	☐	MRI-based neuroimaging

Human research participants

Policy information about studies involving human research participants

Population characteristics	See above.
Recruitment	Participants were recruited through bulletins within the university community. The bulletins mentioned that we are looking for participants for an eye-tracking study on curiosity. Participation was, thus, fully self-selected.
Ethics oversight	Ethics committee of the DIPF | Leibniz Institute for Research and Information in Education

Note that full information on the approval of the study protocol must also be provided in the manuscript.
